# Evaluation of Nidus Occlusion After Radiosurgery in Brain Arteriovenous Malformations—A Prospective Study Using Arterial Spin Labeling

**DOI:** 10.1227/neu.0000000000003590

**Published:** 2025-06-27

**Authors:** Dorian Hirschmann, Wolfgang Marik, Anna Cho, Philippe Dodier, Wei-Te Wang, Arthur Hosmann, Brigitte Gatterbauer, Christian Dorfer, Wolfgang Serles, Lukas Haider, Gregor Kasprian, Josa Maria Frischer

**Affiliations:** * Department of Neurosurgery, Medical University of Vienna, Vienna, Austria;; ‡ Department of Biomedical Imaging and Image-Guided Therapy, Medical University of Vienna, Vienna, Austria;; § Department of Neurology, Medical University of Vienna, Vienna, Austria;; ‖ NMR Research Unit, Queen Square Multiple Sclerosis Centre, Queen Square Institute of Neurology, University College London, London, UK

**Keywords:** Brain arteriovenous malformation, Arterial spin labeling, Radiosurgery, Nidus occlusion, MRI evaluation

## Abstract

**BACKGROUND AND OBJECTIVES:**

The gold standard for the evaluation of brain arteriovenous malformation (AVM) nidus occlusion after stereotactic radiosurgery is digital subtraction angiography (DSA), which is an invasive technique. We evaluated the role of MRI, especially arterial spin labeling (ASL) in the assessment of nidus occlusion after radiosurgery. DSA was used as the gold standard for comparison.

**METHODS:**

Fifty radiosurgically treated brain AVMs were included in this prospective single-center study. All patients underwent a standardized MRI protocol including following sequences: 2-dimensional T2w (TSE) in 3 planes, T1-weighted Magnetization Prepared Rapid Gradient Echo (MPRAGE), axial resolve diffusion-weighted imaging, ASL, time of flight, and time-resolved angiography with interleaved stochastic trajectories. Nidus obliteration according to the standardized MRI protocol was evaluated by an experienced neuroradiologist within 3 days after image acquisition and before DSA was subsequently performed as the reference standard. A second observer retrospectively rated MRI images of all 50 cases blinded to clinical and DSA data after the prospective study was concluded.

**RESULTS:**

All cases rated as obliterated by the MRI protocol were confirmed by DSA. However, 26 and 28 AVMs were rated as patent by the observers, which was verified in 22 (85/79%) cases by DSA. ASL had the highest sensitivity among all MRI sequences. In 3 patients, ASL was the only sequence that correctly revealed a residual nidus according to 1 observer. Overall, the sensitivity and specificity of the standardized MRI protocol for detection of a residual nidus were 100/100% and 86/79%, respectively. The interobserver agreement was excellent (κ = 0.92, 0.81-1.00). At last follow-up of this prospective study, 70% of AVMs were completely obliterated.

**CONCLUSION:**

MRI evaluation of nidus occlusion including ASL is highly sensitive for residual nidus detection and has a high potential to replace invasive DSA examinations for patients who underwent radiosurgery of brain AVMs.

## Graphical abstract

**Figure:**
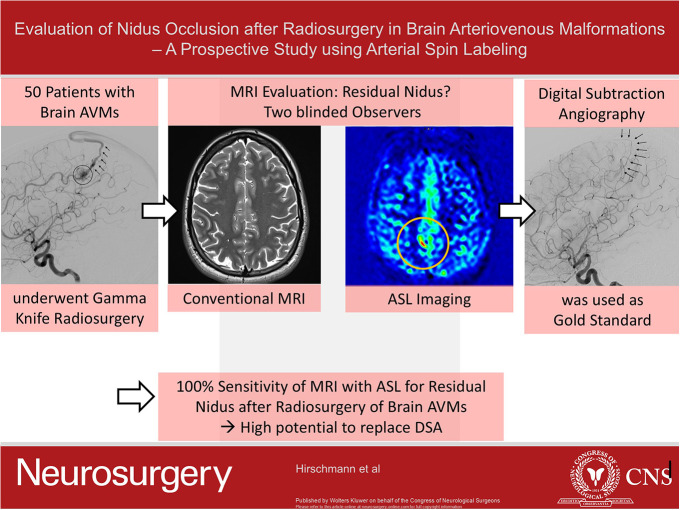

**ABBREVIATIONS:**
ASL: arterial spin labeling
AVF: arteriovenous fistula
CE: contrast-enhanced
MRA: magnetic resonance angiography
NPV: negative predictive value
SM: Spetzler-Martin
SRS: stereotactic radiosurgery
TE: echo time
TOF: time of flight
TR: repetition time
TWIST: time-resolved angiography with interleaved stochastic trajectories.

Complete obliteration is the goal in any arteriovenous malformation (AVM) treatment. Gamma Knife radiosurgery (GKRS) is a well-established, safe, and effective treatment of brain AVMs, either as stand-alone therapy or in combination with prior endovascular embolization^1-6^ Treatment success is time-dependent, and the risk of hemorrhage is not eliminated until complete nidus occlusion.^7^ Hence, reliable evaluation of nidus occlusion is crucial for patients' safety. The gold standard for the evaluation of nidus occlusion is digital subtraction angiography (DSA) which is an invasive method with associated radiation exposure, procedural risks, and high costs. By contrast, MRI is a relatively cost-efficient, noninvasive examination method without radiation exposure.^8^ MRI evaluation of nidus occlusion after stereotactic radiosurgery (SRS) has been reported by numerous authors since 1993. However, in the beginning, sensitivity of conventional MRI sequences did not exceed 80%, and nidus detection was insufficient in AVMs < 1 cm.^9,10^ Since imaging techniques evolved, almost 2 decades thereafter, more specific techniques including magnetic resonance angiography (MRA), such as time-of-flight (TOF) sequences, were reported to reach sensitivities of 84% and beyond.^11-13^ Furthermore, time-resolved angiography with interleaved stochastic trajectories (TWIST) sequences can reveal early enhancement of venous structures.^14^ In 2017 Kodera et al^15^ reported about detection of residual AVM nidi using arterial spin labeling (ASL) MRI in a small series of 7 patients with 100% sensitivity. Initially applied for the evaluation of cerebral perfusion, ASL generates an “endogenous diffusible tracer.” Protons in the arterial blood are labeled by a magnetic pulse at the cervical level and can then be detected intracranially.^16^ According to flow intensity, color-coded images are generated which indicate in vascular malformations areas of increased blood flow representing AV-shunting.^17-20^ In the past years, ASL could further improve noninvasive visualization of treated and untreated AVMs compared with conventional MRA.^14,21-23^ We conducted this prospective, preliminary study to evaluate sensitivity and specificity of a standardized MRI protocol including ASL for the assessment of AVM nidus occlusion after GKRS, using DSA as the reference standard.

## METHODS

### Study Design

Between 2018 and 2024, a consecutive series of patients with an unknown status of AVM nidus occlusion after GKRS treatment at our department was prospectively enrolled in this study. All patients had to give written informed consent to participate, and the minimum age for participation was 16 years. To assess the nidus occlusion status, a standardized MRI protocol as index test was performed after a minimum of 2 years since the last Gamma Knife treatment. Subsequently performed DSA was used as reference standard to evaluate sensitivity and specificity of the MRI protocol.

### Radiosurgical Technique

All patients were treated with Leksell Gamma Knife Perfexion (Elekta AB) at our department. GammaPlan (Elekta AB) was used as planning software. For treatment planning, 3 imaging modalities were performed under stereotactic conditions including MRI with high-resolution T2-weighted scans and TOF magnetic resonance angiography, computed tomography scan with bone window and biplane standard DSA. Planning MRI was performed on either a 1.5 T or 3 T standard scanner, according to patients’ compatibility. As a target, the nidus according to MRI and DSA images without additional margins was used. To minimize the overlapping of radiation dose in volume-staged cases, the nidus was separated creating different volumetric stages as described before.^1^

### Evaluation of Nidus Occlusion

#### Standardized MRI Protocol as Index Test

All patients underwent a standardized MRI protocol. The MRI examination was performed at the earliest 2 years after last GKRS treatment on a Siemens Vida, 3 T scanner with a 64-channel head coil. The protocol included 2-dimensional T2w Turbo Spin Echo (TSE) sequences in 3 planes each with an inplane resolution of 0.2 × 0.2 mm and 2 mm slices thickness with a repetition time (TR) of 5000 ms and an echo time (TE) of 96 ms. Isotropic 1 mm T1-weighted Magnetization Prepared Rapid Gradient Echo (MPRAGE) with TE: 3.28 ms, TR: 2000 ms, inversion time: 1010 ms with and without contrast enhancement. Precontrast and postcontrast T1-weighted images were acquired only for assessment of potential treatment associated changes and were not part of the evaluation of nidus occlusion. Axial resolve diffusion-weighted imaging sequences were acquired with TE: 66 and 114 ms, TR: 5910 ms, b0, b1000, apparent diffusion coefficient map 20 ms, TR: 28 ms, both phase and magnitude images were provided. Pseudo continuous ASL was measured with TE: 17 ms, TR: 4600 ms, with a labeling duration of 1800 ms and a post labeling delay of 1800 ms. TOF sequences with TE: 3.69 ms, TR: 21 ms, with an intracranial field of view 200 mm resolved at 0.3 × 0.3 × 0.5 mm. Time-resolved angiography and contrast-enhanced (CE) TWIST TE: 0.95 ms, TR: 2.55 ms, with a temporal resolution of 1.74 s and without an acquisitions delay. For contrast enhancement, 12 mL of a gadolinium-based contrast agent was used. The acquisition time for the ASL sequence was 4:59 minutes and around 47 minutes for the whole protocol.

#### MRI Analyses

Nidus obliteration according to the standardized MRI protocol was evaluated by 2 experienced neuroradiologists (L. H., W. M.). They separately assessed each sequence as well as the overall protocol for nidus occlusion. Observer 1 prospectively documented MRI results for each case within 3 days after image acquisition and before DSA was performed as reference standard. Observer 2 retrospectively rated MRI images of all 50 cases in 1 session after the prospective study was concluded. Observer 2 was blinded to DSA results and clinical data. Images of the study MRI protocol as well as the treatment planning MRIs of the first GKRS treatment were shown to observer 2 by the study supervisor without any further information or any DSA images. No instructions were given to the observers. The assessment of each sequence and of the overall study protocol was at their discretion.

#### DSA as Reference Standard

Every patient underwent DSA after assessment of the MRI images and documentation of the obliteration status by observer 1. If MRI findings were indicative of a residual nidus, DSA was performed under stereotactic frame-based conditions to enable repeat GKRS treatment in case of DSA confirmation. All DSA images were obtained using Siemens ARTIS icono biplane angiography system. DSA images were assessed by 2 neurosurgeons with specific training in AVM treatment (J. M. F., W. T. W.).

### Data Analyses

The results of the MRI and DSA examinations were compared to calculate sensitivity, specificity, positive predictive value (PPV), and negative predictive value (NPV) for the applied MRI protocol. Calculations were done for both observers separately. For assessment of interobserver variability Cohen κ coefficients including 95% CIs were calculated for each sequence and for the overall protocol. The coefficient was interpreted as follows: κ < 0.45: Poor agreement, 0.45 < κ > 0.75: fair to good agreement, and κ > 0.75: excellent agreement.^23^ Metric variables are presented as counts and percentages. Nonparametric values were used according to the absence of normal distributed data. Sensitivity and specificity as well as PPV and NPV were calculated for the standardized MRI protocol. IBM SPSS Statistics for Windows Version 29.0.2.0 (IBM) was used.

### Ethical Statement

This study was approved by the Institutional Ethics Committee (EK 1497/2018) and complies with the principles of the Declaration of Helsinki. Written informed consent has been obtained from all study participants.

## RESULTS

### Study Population

**FIGURE 1.**
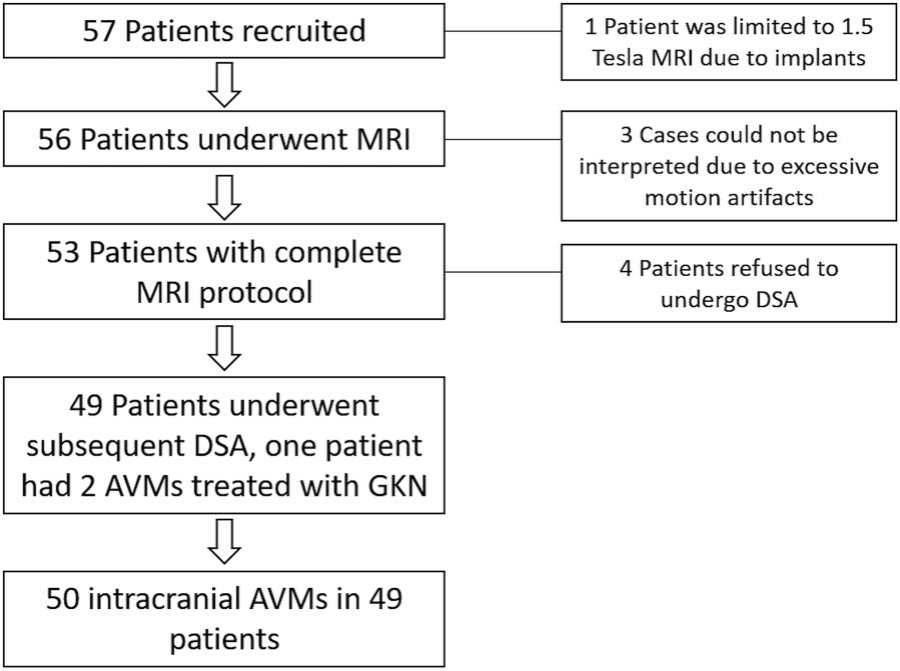
Patient recruitment by flow diagram. AVM, arteriovenous malformation; DSA, digital subtraction angiography.

Patient recruitment is shown in Figure 1. In total, 57 patients were enrolled. One patient could not undergo 3T MRI because of incompatible implants. Thus, 56 patients underwent MRI evaluation according to the protocol. However, 6 patients had to be excluded from the outcome analysis. Reasons were refusal to undergo DSA in 4 cases and insufficient quality of MRI images because of motion artifacts in 2 cases. Hence, 49 patients completed the study. Baseline characteristics of the patients are shown in Table 1. The median patient age was 33.5 (17-69) years, and 52% were male. The median time between MRI and DSA was 51.5 (1-137) days.

**TABLE 1.** Baseline Characteristics of 50 AVM Cases

n = 50	
Median age (min-max)	33.5 (17-69)
Male sex	26 (52%)
AVM location	
Lobes	40 (80%)
Cerebellum	5 (10%)
Basal ganglia/thalamus	3 (6%)
Brainstem	1 (2%)
Midbrain	1 (2%)
Median maximum nidus diameter in cm (min-max)	2.6 (0.7-6.5)
Eloquent region	
Yes	27 (54%)
No	23 (46%)
Venous drainage	
Deep	22 (44%)
Superficial	28 (56%)
Median SM score (min-max)	2 (1-5)
SM Score	
1	8 (16%)
2	22 (44%)
3	14 (28%)
4	5 (10%)
5	1 (2%)
Median number of GKRS treatments (min-max)	1 (1-3)
Median isodose first treatment in % (min-max)	50 (45-50)
Median prescription dose first treatment in Gy (min-max)	19 (18-21)
Median treatment volume first treatment in ccm (min-max)	2.6 (0.2-11.4)
Median isodose last treatment in % (min-max)	50 (45-60)
Median prescription dose last treatment in Gy (min-max)	19 (16-20)
Median treatment volume last treatment in ccm (min-max)	1.65 (0.1-9.9)
Prior embolization	
Yes	19 (38%)
No	31 (62%)
Prior microsurgical resection	0
Prior hemorrhage	25 (50%)
Median time between last GKRS and MRI in months (min-max)	28 (22-204)
Median time between MRI and DSA in days (min-max)	51.5 (1-137)

AVM, arteriovenous malformation; DSA, digital subtraction angiography; GKRS, Gamma Knife Radiosurgery; Gy, gray; MRI, Magnetic resonance imaging; SM, Spetzler-Martin.

### AVM and Patient Characteristics

Of the 49 patients, 1 had 2 AVMs treated by GKRS; hence, in total 50 AVMs were evaluated. AVM characteristics are depicted in Table 1. Most AVMs were located in the lobes of the cerebrum, and the median diameter of the nidus was 2.6 (0.7-6.5) cm. The venous drainage was deep in 44%, and localization was eloquent in 54%. The median Spetzler-Martin (SM) score was 2 (1-5). The median number of GKRS treatments was 1 (1-3). Treatment planning mainly occurred on the 50% isodose with a median prescription dose of 19 Gy. The median time between last GKRS treatment and MRI examination was 28 (22-204) months. One-third of the patients had undergone partial embolization before GKRS, and half of the patients had suffered from prior hemorrhage.

### Evaluation of Nidus Occlusion

Of 50 AVMs, the nidus was rated occluded in 24 cases by observer 1 and 22 cases by observer 2. Consequently, 26 and 28 cases were rated patent according to MRI, respectively (Table 2). In all cases in which complete obliteration was diagnosed on MRI by either of the observers, this finding was verified by DSA. By contrast, of the 26 (observer 1) and 28 (observer 2) cases rated as patent on MRI, the AVM was confirmed as obliterated via DSA in 4 cases and 6 cases, respectively. Hence, sensitivity of the standardized MRI protocol for detection of a residual nidus was 100% for both observers. Specificity was 86% (observer 1) and 79% (observer 2), respectively. Consequently, the PPV of the overall MRI protocol for residual nidus were 85% (observer 1) and 79% (observer 2) and the NPV was 100% for both observers. The interobserver agreement for the overall protocol was excellent (κ = 0.92, 0.81-1.00).

**TABLE 2.** AVM Occlusion Status According to Each MRI Sequence as Well as Overall MRI Result in Comparison With DSA as a Reference

MRI diagnosis	DSA diagnosis		Cohen κ	Total
	Residual nidus	Obliterated		
T2 observer 1/2				
Residual nidus	17/20 **(77%/91%)**	7/4 (25%/14%)	0.60 (0.38-0.82)	24/24
Obliterated	5/2 (23%/9%)	21/24 **(75%/86%)**		26/26
TOF observer 1/2				
Residual nidus	15/18 **(68%/82%)**	2/4 (7%/14%)	0.63 (0.41-0.84)	17/22
Obliterated	7/4 (32%/18%)	26/24 **(93%/86%)**		33/28
TWIST^a^ observer 1/2				
Residual nidus	16/16 **(76%/76%)**	2/1 (7%/5%)	0.78 (0.60-0.96)	18/17
Obliterated	5/5 (24%/24%)	26/27 **(93%/95%)**		31/32
ASL^b^ observer 1/2				
Residual nidus	21/21 **(95%/95%)**	4/6 (15%/21%)	0.88 (0.75-1.00)	25/27
Obliterated	1/1 (5%/5%)	23/22 **(85%/79%)**		24/23
Overall MRI diagnosis observer 1/2				
Residual nidus	22/22 **(100%/100)**	4/6 (14%/21%)	0.92 (0.81-1.00)	26/28
Obliterated	0/0	24/22 **(86%/79%)**		24/22

ASL, arterial spin labeling; AVM, arteriovenous malformation; DSA, digital subtraction angiography; TOF, time of flight; TWIST, time-resolved angiography with interleaved stochastic trajectories.

a One case of TWIST had to be excluded because of motion artifacts.

b One ASL sequence was rated as uninterpretable by observer 1 but obliterated by observer 2.

All cases of patent AVMs were diagnosed by MRI, resulting in a 100% sensitivity of the overall standardized protocol for the detection of a residual nidus.

Bold numbers represent sensitivity and specificity.

Detailed results of the MRI sequences compared with those of DSA are shown in **Supplemental Digital Content 1** (http://links.lww.com/NEU/E855). Depending on the observer, the stand-alone sensitivities for T2, TOF, TWIST, and ASL were 77/91%, 68/82%, 76/76%, and 95/95%, respectively. The corresponding specificities were 75/86%, 93/86%, 93/95%, and 85/79%. The interobserver agreement was good for T2 and TOF sequences and excellent for TWIST and ASL (see Table 2). Of note, 3 cases were diagnosed with residual nidus correctly only according to the ASL sequence by observer 1 because they were rated obliterated on T2, TOF, and TWIST. Hence, ASL was the most sensitive sequence for detection of a residual nidus. However, quality of ASL images was rated insufficient in 1 case by observer 1, and TWIST was rated of insufficient quality in another case by both observers because of motion artifacts.

### Obliteration Rates

Long-term radiological results are shown in **Supplemental Digital Content 2** (http://links.lww.com/NEU/E856). At the time of study conclusion, the overall nidus occlusion rate was 70% (35/50). Of note, obliteration status at the time of last follow-up was significantly associated with SM grade (*P* = .002). Hence, of all patients in which total obliteration had not been achieved at study conclusion, 80% (12/15) were SM grade 3 or higher. For these patients, further GKRS treatments were planned outside of the study inclusion period.

### Illustrative Case 1

**FIGURE 2.**
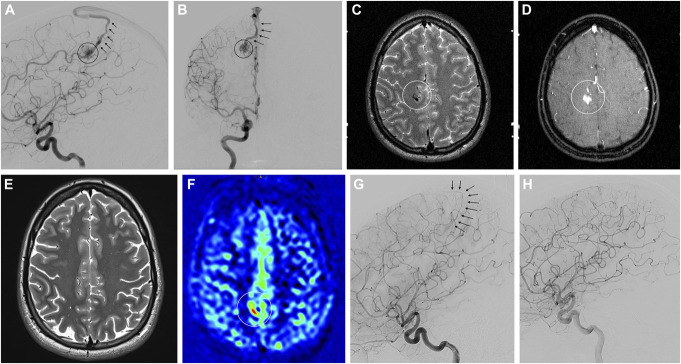
**A** and **B**, Lateral and frontal angiograms of a Spetzler-Martin 2 AVM in the right central region after right internal artery injection (circle = nidus, arrows = draining vein). The nidus was also seen on **C**, T2-weighted and **D**, time-of-flight MRI images (circle). Two years after Gamma Knife radiosurgery, the AVM was rated obliterated on **E**, T2w images, **F**, but arterial spin labeling indicated persisting AV-shunting (circle). **G**, An early draining vein was seen on the angiogram (arrows). However, 1 year later, **H**, the AVM was proven to be totally obliterated by repeat angiography without further treatment. AVM, arteriovenous malformation.

A patient in their 20s had undergone single GKRS treatment of a SM 2 AVM in the right central region (Figure 2A-2D). The standardized MRI protocol was performed 115 weeks after GKRS and T2, TOF, and TWIST sequences were rated obliterated (T2 sequence shown in Figure 2E). By contrast, ASL indicated a residual AV-shunt compatible with the initial draining vein (Figure 2F). A DSA without stereotactic frame was performed which confirmed an early draining vein (Figure 2G) but no additional treatment was performed. Follow-up DSA which was performed 1 year later showed complete obliteration of the AVM without visualization of the early draining vein (Figure 2H).

### Illustrative Case 2

**FIGURE 3.**
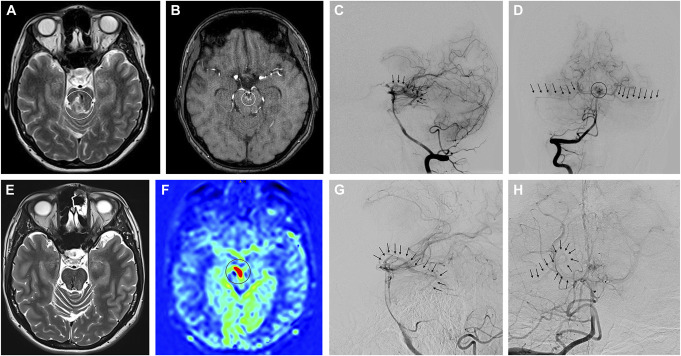
After suffering from hemorrhage (circle) of a pontine AVM (**A**, T2-weighted MRI, **B**, time of flight, circle = nidus), a patient in their 20s underwent DSA and GKRS. **C** (lateral view) and **D** (frontal view), Pre-GKRS angiograms after right vertebral artery injection (arrows = early draining veins, circle = nidus). In the standardized MRI protocol after 2 years, **E**, the AVM was rated obliterated according to T2 images, **F**, but AV-shunting (circle) was seen on arterial spin labeling. Hence, DSA was performed and the patient underwent retreatment. An early draining vein (arrows) is seen on **G**, lateral and **H**, frontal angiograms. AVM, arteriovenous malformation; DSA, digital subtraction angiography; GKRS, Gamma Knife radiosurgery.

Another patient in their 20s suffered from a symptomatic hemorrhage in the pons caused by a small AVM SM grade 3 (Figure 3A and 3B). The patient was admitted to the intensive care unit and underwent DSA (Figure 3C and 3D). Endovascular embolization was abandoned because of the estimated high risk of perioperative morbidity. Three months later, GKRS was performed. The standardized MRI protocol which was performed 120 weeks after treatment did not show any residual nidus except for the ASL sequence which indicated a residual nidus (Figure 3E and 3F). Subsequent DSA under stereotactic conditions confirmed this finding, and retreatment was performed (Figure 3G and 3H). The patient recovered without any neurological sequelae.

### Illustrative Case 3

**FIGURE 4.**
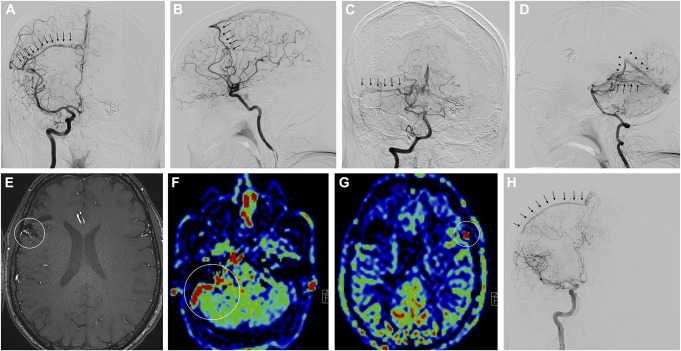
This patient underwent GKRS of a right frontal partially embolized AVM. **A** and **B**, Frontal and lateral internal carotid angiograms (arrows = early draining vein). **C** and **D**, Frontal and lateral angiograms of a ruptured AVM of the tectum after vertebral artery injection (arrows = early draining vein). **E**, MRI 121 weeks after GKRS showed persistence of the right frontal AVM nidus (circle) in time of flight, **F** and **G**, but in ASL only the right tectal AVM and the left frontal arteriovenous fistula were seen (circles). **H**, DSA confirmed early filling of the right frontal draining vein (arrows) after right internal carotid injection which was not seen on ASL. This was the only case of false-negative ASL. However, the overall result of the MRI protocol was true positive. ASL, arterial spin labeling; AVM, arteriovenous malformation; GKRS, Gamma Knife radiosurgery.

An adult patient suffered from an intracerebral hemorrhage of an AVM in the right tectum. DSA furthermore revealed a right frontal AVM and a left frontal dural arteriovenous fistula (AVF). After embolization of the AVF and the right frontal AVM, the patient underwent GKRS treatment of the 2 AVMs (Figure 4A-4D). After 121 weeks, persistence of the right frontal AVM in TOF images was seen (Figure 4E). However, ASL was only indicative of patency of the AVM located in the right tectum and of the left frontal AVF (Figure 4F+4G). DSA confirmed a right frontal residual nidus (Figure 4H). This was the only case of a false-negative result of an ASL sequence. Nonetheless, both AVMs were rated patent in the MRI protocol which thus, resulted in a true-positive overall finding.

## DISCUSSION

### Evaluation of a Standardized MRI Protocol

In this study, the standardized MRI protocol which assessed nidus occlusion of 50 AVMs after GKRS had a NPV of 100% according to both observers. Consequently, in all cases which were rated occluded according to MRI, occlusion was confirmed by DSA. Furthermore, the interobserver agreement for the overall protocol was excellent. Because radiological findings may vary between radiologists, interobserver agreement is crucial for reproducibility. Among all single sequences, ASL had the highest interobserver agreement. These findings correspond well to those of others, who reported good interobserver agreement for TOF and excellent agreement for ASL.^11,18,23^ When nidus occlusion status of an AVM is assessed after GKRS by MRI, the most important parameter is sensitivity for residual nidus. A residual nidus which is not identified on MRI leads to a false-negative result which means the patient is still at risk of hemorrhage.^24,25^ In the 50 prospectively evaluated cases of this, study there were no false-negative results. On the contrary, 4 cases in which the nidus was occluded were rated patent on MRI, which led to a false-positive result. However, these patients are not at risk of hemorrhage, thus such findings are not as relevant as false negatives. In the past, MRI assessment of nidus occlusion after GKRS has been evaluated by others who reported lack of visualization in case of a nidus <1 cm.^9^ Furthermore, according to Lee et al^12^ the sensitivity of MRI to detect a residual AVM nidus after GKRS was between 76.7% and 84.9%. However, those studies did not use ASL or TWIST sequences.

### The Role of ASL

ASL sequences were the most sensitive for the assessment of nidus occlusion in our study. In 3 cases of a patent AVM nidus, ASL was the only MRI sequence to indicate persistent AV-shunting according to 1 observer. Although ASL perfusion does not provide high structural resolution, it can visualize AV-shunting effectively.^17^ Hence, its use for the assessment of AVM nidus occlusion after GKRS seems obvious. Heit et al^18^ reported a 100% sensitivity and 95% specificity of ASL for residual nidus detection after SRS in a retrospective study which included 15 cases using ASL. A prospective study by Kodera et al^15^ identified all cases of residual AVM nidus after SRS using ASL imaging in a small cohort of 7 patients. Consequently, ASL has been proposed as a promising method for the evaluation of nidus occlusion after SRS.^17^

During the conduction of our study, 2 other studies have prospectively evaluated the sensitivity and specificity of ASL imaging for the detection of persistent AVM nidus after GKRS compared with DSA: Rojas-Villabona et al^26^ reported 100% sensitivity and 100% specificity of a MRI protocol including ASL MRA and CE time-resolved MRA in 30 patients. Furthermore, Leclerc et al found superiority of ASL/TOF compared to conventional MRI sequences in a cohort of 28 patients. Sensitivity and specificity for detection of residual nidus were 85% and 100% compared with 55% and 100% in conventional CE sequences. It has to be mentioned that in the latter study, observers were blinded to the site of the AVM and thus, were prone to discard minor signal alterations on ASL imaging.^23^ Contrary to this method, observers in the study conducted by Rojas-Villabona et al as well as in our study had access to preexisting images to simulate a conventional clinical follow-up scenario. This difference in methodology may be responsible for the reduced sensitivity in the study by Leclerc et al.

Of note, similar to our study, Leclerc et al used ASL perfusion imaging, whereas Rojas-Villabona et al used ASL angiography. The encouraging results of Leclerc et al and Rojas-Villabona et al could be confirmed in our study, which is the largest to date including 50 AVM cases.

In only 1 case of our study, AV-shunting was not detected by ASL. The reason remains unclear; however, possible mechanisms may include prior treatment by embolization with onyx and coexistence of a contralateral pial AV-fistula, as well as an AVM in the posterior fossa.

### Limitations

Although this is the first prospective study to compare MRI with DSA after GKRS of AVMs in 50 cases, the case number is still limited. Hence, the results must be verified by a larger series, preferably in a multicenter approach. Despite an excellent interobserver agreement, interpretation of MRI images may vary between different observers or may be dependent of experience and capabilities. Neurosurgeons who performed and assessed DSA were not blinded to MRI results because the DSA functioned as the reference standard.

## CONCLUSION

A comprehensive MRI protocol including ASL achieves a 100% sensitivity for the detection of a residual AVM nidus. Thus, a standardized MRI evaluation of nidus occlusion by trained neuroradiologists could potentially replace invasive DSA examinations in patients who underwent GKRS for an intracranial AVM.

## Supplementary Material

## SUPPLEMENTAL DIGITAL CONTENT

**Supplemental Digital Content 1**. **Table 1**. Shows the results according to both observers of each single MRI sequence in our protocol and the final diagnosis and evaluation according to the angiography (*missing result due to limited quality according to reviewer). + indicates patent AVM/− indicates occluded AVM. **Supplemental Digital Content 2**. **Table 2**. Of 50 patients, in 35 (70%) complete obliteration was documented by angiography at time of last follow-up in our prospective study. Obliteration status was significantly dependent on Spetzler-Martin grade (*P* = .002).
