# Supplementary material for: Evaluation of Nidus Occlusion After Radiosurgery in Brain Arteriovenous Malformations—A Prospective Study Using Arterial Spin Labeling
Source: Neurosurgery. 2025 Jun 27;98(1):96–104. doi: 10.1227/neu.0000000000003590 (PMC12680279; doi:10.1227/neu.0000000000003590)
Supplement: Supplementary file 1 [file neu-98-096-s001.docx]

Supplementary table 1 shows the results of each single MRI sequence in our protocol and the final diagnosis and evaluation according to the angiography (*missing result due to limited quality according to observer). + indicates patent AVM / - indicates occluded AVM.

| n=50 | **AVM localization** | **Prior hemorrhage** | **Prior embolization** | **T2**  **Obs. 1/2** | **TOF**  **Obs.1/2** | **TWIST**  **Obs.1/2** | **ASL**  **Obs.1/2** | **MRI overall**  **Obs. 1/2** | **DSA** | **Result MRI vs DSA**  **Observer 1/2** |
| --- | --- | --- | --- | --- | --- | --- | --- | --- | --- | --- |
|  |  |  |  |  |  |  |  |  |  |  |
| 1 | Parieto-occipital lobes | No | Yes | -/- | -/- | -/- | -/- | -/- | - | true negative/  true negative |
| 2 | Occipital lobe | Yes | No | +/- | +/- | -/- | +/+ | +/+ | + | true positive/  true positive |
| 3 | Occipital lobe | No | No | +/+ | +/+ | */* | +/+ | +/+ | + | true positive/  true positive |
| 4 | Occipital lobe | Yes | No | +/- | -/+ | -/+ | +/+ | +/+ | - | false positive/  false positive |
| 5 | Temporal lobe | No | No | +/+ | +/+ | +/+ | +/+ | +/+ | + | true positive/  true positive |
| 6 | Temporal lobe | No | No | +/+ | +/+ | +/+ | +/+ | +/+ | + | true positive/  true positive |
| 7 | Brainstem | Yes | No | -/- | -/+ | -/+ | +/+ | +/+ | + | true positive/  true positive |
| 8 | Fronto-parietal lobes | No | No | -/- | -/- | -/- | -/- | -/- | - | true negative/  true negative |
| 9 | Frontal lobe | Yes | Yes | -/- | -/- | -/- | -/- | -/- | - | true negative/  true negative |
| 10 | Frontal lobe | Yes | Yes | -/- | -/- | -/- | -/- | -/- | - | true negative  true negative |
| 11 | Occipital lobe | No | No | -/- | -/- | -/- | -/- | -/- | - | true negative/  true negative |
| 12 | Parietal lobe | Yes | Yes | -/- | +/- | -/- | -/- | -/- | - | true negative/  true negative |
| 13 | Fronto-parietal lobes | No | No | -/+ | -/- | -/- | +/+ | +/+ | + | true positive/  true positive |
| 14 | Cerebellum | Yes | No | -/- | -/- | -/- | -/- | -/- | - | true negative/  true negative |
| 15 | Fronto-parietal lobes | Yes | No | -/- | -/- | -/- | -/- | -/- | - | true negative/  true negative |
| 16 | Cerebellum | Yes | Yes | +/+ | +/+ | -/- | -/+ | -/+ | - | true negative/  false positive |
| 17 | Cerebellum | Yes | Yes | -/+ | -/- | +/- | +/+ | +/+ | - | false positive/  false positive |
| 18 | Parietal lobe | No | No | +/+ | +/+ | +/+ | +/+ | +/+ | + | true positive/  true positive |
| 19 | Parietal lobe | No | No | +/- | -/- | -/- | -/- | -/- | - | true negative/  true negative |
| 20 | Parietal lobe | No | No | +/+ | +/+ | +/+ | +/+ | +/+ | + | true positive/  true positive |
| 21 | Fronto-parietal lobes | Yes | No | +/+ | +/+ | +/+ | +/+ | +/+ | + | true positive/  true positive |
| 22 | Fronto-parietal lobes | No | No | -/- | -/- | -/- | -/- | -/- | - | true negative/  true negative |
| 23 | Fronto-parietal lobes | No | No | +/+ | -/- | -/- | +/+ | +/+ | - | false positive/  false positive |
| 24 | Temporal lobe | Yes | Yes | +/+ | +/+ | +/+ | +/+ | +/+ | + | true positive/  true positive |
| 25 | Frontal lobe | Yes | Yes | +/+ | -/- | -/- | +/+ | +/+ | + | true positive/  true positive |
| 26 | Temporal lobe | No | Yes | +/+ | -/+ | +/+ | +/+ | +/+ | + | true positive/  true positive |
| 27 | Fronto-parietal lobes | No | No | -/+ | -/- | -/- | +/+ | +/+ | + | true positive/  true positive |
| 28 | Cerebellum | Yes | Yes | -/+ | -/+ | -/- | -/- | -/- | - | true negative/  true negative |
| 29 | Occipital lobe | Yes | No | +/- | -/- | +/- | -/+ | -/+ | - | true negative/  false positive |
| 30 | Basal ganglia | Yes | No | +/- | +/+ | +/+ | +/+ | +/+ | + | true positive/  true positive |
| 31 | Occipital lobe | Yes | No | -/- | -/+ | +/+ | +/+ | +/+ | + | true positive/  true positive |
| 32 | Thalamus | Yes | Yes | -/- | -/- | -/- | -/- | -/- | - | true negative/  true negative |
| 33 | Temporal lobe | No | No | -/- | -/- | -/- | -/- | -/- | - | true negative/  true negative |
| 34 | Parietal lobe | No | Yes | +/+ | +/+ | +/+ | +/+ | +/+ | + | true positive/  true positive |
| 35 | Occipital lobe | No | No | +/- | -/- | -/- | -/- | -/- | - | true negative/  true negative |
| 36 | Fronto-parietal lobes | No | No | -/- | -/- | -/- | -/- | -/- | - | true negative/  true negative |
| 37 | Parietal lobe | No | No | +/- | -/+ | -/- | -/- | -/- | - | true negative/  true negative |
| 38 | Temporo-occipital lobes | No | No | -/- | -/+ | -/- | -/- | -/- | - | true negative/  true negative |
| 39 | Temporal lobe | Yes | Yes | -/- | -/+ | -/- | +/+ | +/+ | - | false positive/  false positive |
| 40 | Frontal lobe | Yes | Yes | -/- | -/+ | -/- | -/- | -/- | - | true negative/  true negative |
| 41 | Fronto-Parietal lobes | Yes | No | +/+ | +/+ | +/+ | +/+ | +/+ | + | true positive/  true positive |
| 42 | Thalamus | Yes | Yes | -/- | -/- | -/- | -/- | -/- | - | true negative/  true negative |
| 43 | Fronto-parietal lobes | No | No | +/+ | -/+ | +/+ | +/+ | +/+ | + | true positive/  true positive |
| 44 | Frontal lobe | No | No | -/- | -/- | -/- | -/- | -/- | - | true negative/  true negative |
| 45 | Midbrain | Yes | No | -/+ | +/+ | +/- | +/+ | +/+ | + | true positive/  true positive |
| 46 | Frontal lobe | No | Yes | +/+ | +/+ | +/+ | -/- | +/+ | + | true positive/  true positive |
| 47 | Parieto-occipital lobes | No | Yes | +/+ | +/+ | +/+ | +/+ | +/+ | + | true positive/  true positive |
| 48 | Fronto-parietal lobes | Yes | No | +/+ | +/+ | +/+ | +/+ | +/+ | + | true positive/  true positive |
| 49 | Cerebellum | Yes | Yes | -/- | -/- | -/- | */- | -/- | - | true negative/  true negative |
| 50 | Frontal lobe | No | Yes | -/- | -/- | -/- | -/- | -/- | - | true negative/  true negative |
