# Supplementary material for: Evaluation of Nidus Occlusion After Radiosurgery in Brain Arteriovenous Malformations—A Prospective Study Using Arterial Spin Labeling
Source: Neurosurgery. 2025 Jun 27;98(1):96–104. doi: 10.1227/neu.0000000000003590 (PMC12680279; doi:10.1227/neu.0000000000003590)
Supplement: Supplementary file 2 [file neu-98-096-s002.docx]

Supplementary table 2

Of 50 patients, in 35 (70%) complete obliteration was documented by angiography at time of last follow-up in our prospective study. Obliteration status was significantly dependent on Spetzler-Martin grade (p=0.002).

| Overall Result | SM 1 | SM 2 | SM 3 | SM 4 | SM 5 |  | Total |
| --- | --- | --- | --- | --- | --- | --- | --- |
|  |  |  |  |  |  |  |  |
| Occluded | 7 (20%) | 20 (57%) | 6 (17%) | 1 (3%) | 1 (3%) |  | 35 (70%) |
|  |  |  |  |  |  |  |  |
| Patent | 1 (7%) | 2 (13%) | 8 (53%) | 4 (27%) | 0 |  | 15 (30%) |
|  |  |  |  |  |  |  |  |
|  |  |  |  |  |  |  | 50 |
